# Kidney morphology in pregnancy using T2-weighted MRI

**DOI:** 10.1016/j.crad.2022.03.019

**Published:** 2022-05-13

**Authors:** F. Conti-Ramsden, R. Hill, M. Rutherford, N. Railton, L.C. Chappell, K. Wiles

**Affiliations:** a Department of Women and Children’s Health, School of Life Course Sciences, King’s College London, London, UK; b Centre for the Developing Brain, King’s College London, London, UK; c Department of Radiology, Mid and South Essex NHS Foundation Trust, UK; d Departments of Renal Medicine and Women’s Health, Barts Health NHS Trust, London, UK

## Abstract

**Aim:**

To report the morphology of maternal kidneys captured on fetal magnetic resonance imaging (MRI) including kidney length, volume, renal pelvis diameter, and corticomedullary differentiation in pregnancy.

**Materials and Methods:**

A retrospective study of maternal kidney morphology captured incidentally on fetal MRI. Women without chronic kidney disease, with a complete view of both kidneys and a singleton pregnancy were included. Kidney length, maximal renal pelvis diameter, kidney volume, and corticomedullary differentiation ratio were measured independently in duplicate. Associations with maternal and pregnancy variables were explored using linear regression.

**Results:**

MRI images from 42 women were performed at 22–32 weeks’ gestation. Serum creatinine concentrations are not checked routinely during pregnancy and were available for 15 (36%) women, with a median creatinine of 57 µmol/l (IQR: 50–63 µmol/l). Mean interpolar lengths were 10.9 and 10.4 cm for the left and right kidneys and varied with height. Mean maximal renal pelvis diameters were 9 mm and 12 mm, with upper reference intervals of 17 and 25 mm for the left and right kidneys, respectively. Renal volume in pregnancy was within the non-pregnant reference interval and varied with height and gestation.

**Conclusions:**

Maternal kidney length and volume in pregnancy are within the normal reference intervals for non-pregnant women. Renal pelvis diameter in pregnancy measured using MRI is substantially higher than described previously by ultrasound, with implications for routine reporting.

## Introduction

Substantial changes are reported in the morphology of the maternal kidney and renal tract in pregnancy based on historical plain X-ray film and ultrasound data. These include an increase in kidney length by 1 cm^1^ and a 30% increase in mean kidney volume to 292 ml.^2^ Gestational dilatation of the renal pelvis is also described, with upper reference limits (97.5^th^ centile) of 12 and 16 mm in the left and right kidneys, respectively.^3^

Magnetic resonance imaging (MRI) is increasingly used as a diagnostic technique in pregnancy,^4^ and is an established fetal imaging technique.^5^ Outside of pregnancy, MRI has been shown to be more accurate than ultrasound in determining kidney and renal tract morphology,^6,7^ as well as being used for functional investigation of the kidneys.^8^

There is a need for contemporary and accurate kidney and renal tract morphology data in healthy pregnancy in order to distinguish physiological gestational change from pathology. Reference intervals for kidney morphology on MRI are required as increasing numbers of MRI examinations in pregnancy are performed for both maternal and fetal indications. The present study reports the morphology of maternal kidneys captured on fetal MRI, including kidney length, volume, renal pelvis diameter, and corticomedullary differentiation in pregnancy.

## Materials and methods

MRI and clinical data were obtained from the iFind-2 study (http://www.ifindproject.com/), which aims to improve antenatal fetal abnormality detection. In this study, healthy pregnant women and women with suspected or confirmed fetal abnormalities consent to undergo fetal ultrasound and MRI in pregnancy. iFind-2 study participants consent to the use of their images and anonymised clinical data for additional research purposes, approved by the Research Ethics Committee and Health Research Authority (14/LO/1806).

MRI examinations of iFind-2 study participants over a 15-month period (January 2016 to March 2017) were reviewed for inclusion in this study. Images were included from women with healthy singleton pregnancies where a complete view of both maternal kidneys was available on MRI. Women with known maternal kidney disease and women with diagnosed or suspected fetal abnormalities on routine ultrasound were not included. Demographic and clinical data were obtained from the iFind-2 study database and from anonymised, retrospective data collected as part of usual patient care by the direct-care clinical team.

MRI examinations were performed on a 1.5 T Philips Achieva MRI machine using a T2-weighted echo acquisition sequence centred on the pregnant uterus. Images used for renal morphology measurements were obtained with the following sequence parameters: 90 ms echo time (TE), 921 ms repetition time (TR), SE scan mode, SENSitivity Encoding (SENSE), 2.5 mm section thickness. All iFind-2 MRI examinations were reported by a perinatal radiologist, including both incidental maternal and fetal findings.

**Figure 1:**
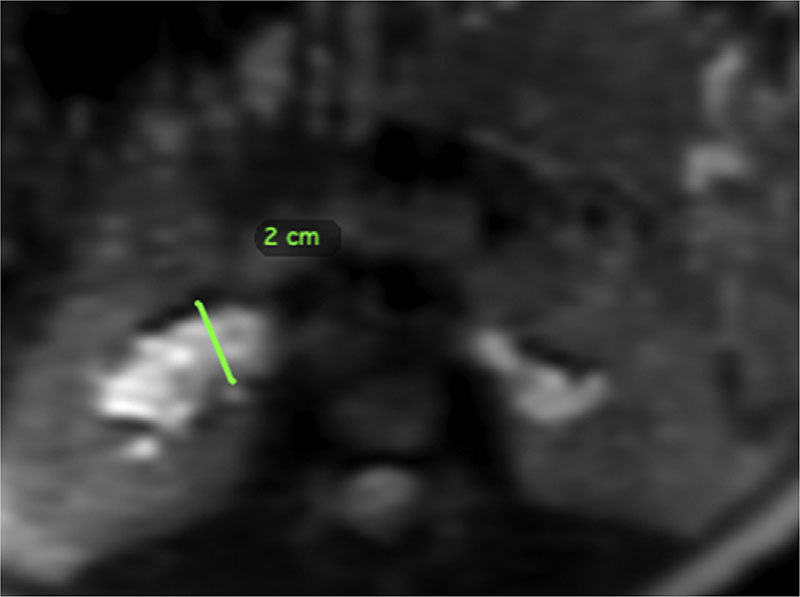
Reformatted transverse view of maximum renal pelvis diameter.

**Figure 2:**
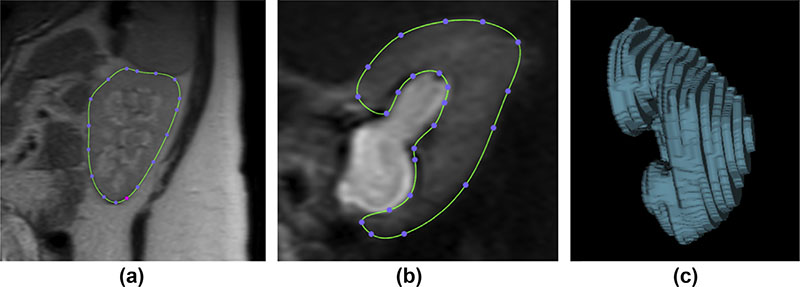
Illustration of disc summation kidney volume estimation method. Manual contour tracing of kidneys on each available MRI section was undertaken (a) with exclusion of renal pelvis and vasculature (b), to generate a rendered reconstructed three-dimensional image (c) from which volume was derived.

Maternal kidney and renal pelvis morphology were assessed independently by two clinicians using OsiriX software.^9^ Kidney length was defined as the maximal interpolar length on a single MRI section. Maximum renal pelvis diameter was measured on a transverse view of the kidney (Fig 1), provided reconstructed images were of sufficient quality to allow measurement. Kidney parenchymal volume was calculated using disc summation^10^ in which the contour of the kidney (excluding the renal pelvis and vasculature) was traced on each MRI section. Areas were then summed across all sections and multiplied by section thickness to generate kidney volume (see Fig 2). Cortico-medullary differentiation was quantified using the section where differentiation between the cortex and medulla of the kidneys was most evident by visual inspection. Mean signal intensity in the cortex and medulla was derived from three, non-overlapping circular region of interest samplers (5–10 mm^3^) and a ratio of mean cortex:medulla signal intensity generated.

### Statistical analysis

Mean kidney length, volume, and renal pelvis diameter were calculated using combined data from both observers. Reference intervals were calculated using robust methods.^11^ Differences in measurements between the left and right kidney were assessed using Student’s t-test. Interobserver variability of measurements was assessed using BlandeAltman plots and computation of Lin’s concordance correlation coefficients.^12^ Linear regression with complete case analysis was used to explore the association between renal morphology and maternal and pregnancy variables. All data handling and analysis was performed in R version 3.6.3.^13^

## Results

Forty-two MRI examinations performed in pregnant women were included. The MRI examinations were performed predominantly within the second trimester at a median gestation of 26 weeks’ gestation (interquartile range (IQR): 23.7-27.6 weeks, range 22.1-31.9 weeks). Most women were of White European ethnicity. Demographic and pregnancy outcome data are shown in Table 1.

Serum creatinine concentrations during pregnancy were available for 15 (35.7%) women. Serum creatinine had been checked prior to the MRI examination in three women and after the MRI examination in 12 women at a median gestational age of 37.2 weeks (range 29-40.4 weeks). Median serum creatinine was 57 µmol/l (IQR: 50-63 µmol/ l) and the maximum serum creatinine was 68 µmol/l, which is within the normal reference interval for pregnancy.^14^ There was no clinical indication to check serum creatinine in the reminder, according to usual pregnancy practice. There was an additional single post-partum serum creatinine concentration available for one woman, which was 62 µmol/l.

Fifteen women (35.7%) had incidental maternal abnormalities reported on the MRI. Visible maternal renal pelvis dilatation was reported in eight (19.0%) women, including unilateral dilatation on the right in five (11.9%) women and bilateral renal pelvis dilatation in three (7.1%) women. There was no specific renal pelvis diameter threshold at which dilatation was reported. Unilateral simple renal cysts were seen in four (9.5%) women, the largest of which measured 11 mm. Bilateral small sub-centimetre cysts were seen in one woman. One woman had a duplex collecting system on the left side, which was excluded from renal pelvis diameter measurement in this study. The remaining incidental abnormalities were outside of the kidney and renal tract and included a sacral Tarlov cyst, intervertebral disc degeneration, and liver and cervical cysts.

Abnormalities, which had not been seen on routine ultrasound imaging in pregnancy, were detected on MRI in five fetuses. These included borderline ventriculomegaly, asymmetrical lateral ventricles, a prominent cisterna magna, a dolichocephalic skull, and a small orbital cyst. These were managed through local clinical reporting protocols.

Reconstructed transverse images were of sufficient quality to allow to allow measurement of the renal pelvis diameter in 38 kidneys on the left and 37 kidneys on the right. Corticomedullary differentiation was measurable in 76 (90.5%) kidneys, including 42 (100%) right kidneys and 34 (80.9%) left kidneys. There was excellent concordance between observer measurements of interpolar length, renal pelvis diameter, and kidney volume (Table 2).

Renal morphology measurements on MRI in pregnancy are shown in Table 2. Renal pelvis diameter was substantially bigger on the right compared to the left. Renal pelvis diameter was >8 mm on the left in 45% and >20 mm on the right in 8%. There was no difference in maximal interpolar length, kidney volume, or corticomedullary differentiation between the left and right kidneys. Maximum interpolar length was associated with maternal height. Kidney volume was associated with both maternal height and gestational age, with a measurable increase in kidney volume for each week of advancing gestation within the second trimester (Table 3).

## Discussion

Kidney and renal tract morphology in pregnancy can be assessed using MRI, with good concordance between observers. Mean kidney interpolar lengths in pregnancy are 10.4 cm and 10.9 cm for the right and left kidney, respectively, with variance according to maternal height. The renal pelvis dilates in pregnancy with mean diameters of 9 and 12 mm and suggested upper reference limits of 17 and 25 mm for the left and right kidneys, respectively, in this cohort. Kidney volume can be measured on MRI and varies according to maternal height and gestational age within the second trimester. Assessment of corticomedullary differentiation is feasible on MRI, although interobserver concordance is low.

To the authors’ knowledge, this is the first study to report normal renal morphology in pregnancy on MRI. The mean maximum interpolar lengths reported are within the normal reference intervals for kidney length reported for non-pregnant women on MRI where mean length is 11.6 cm, and upper reference limit (mean plus two standard deviations) is 13.8 cm.^10^ Kidney length reported here is also comparable to non-pregnant values derived from ultrasound images,^15^ as well as previous studies reporting that the left kidney tends to be slightly longer than the right kidney.^15,16^ Kidney length reported here disputes the commonly cited finding that kidney size increases by 1–1.5 cm in pregnancy, a conclusion derived from small, historical cohorts using ultrasound^17^ and plain-film contrast imaging in the post-partum period.^1^ Although this study was an opportunistic analysis of the maternal kidney captured on fetal MRI, rather than a targeted image of the kidney, and sections may not have captured the true maximal renal length, it seems plausible that kidney length does not substantially increase in pregnancy, contrary to established teaching.

The finding of increased renal pelvis diameter in pregnancy is consistent with the known physiological changes of pregnancy, which include progressive dilatation of the renal pelvis, calyces, and ureters, thought to occur due to progesterone-induced reductions in smooth muscle tone and peristalsis, in conjunction with mechanical compression of the ureters by the gravid uterus. Renal pelvis dilatation is recognised to be more prominent on the right, with dextrorotation of the uterus by the sigmoid colon and kinking of the ureter as it crosses the right iliac artery hypothesised contributing factors.^18^ Ureteric dilatation of up to 8 mm on the left and 20 mm on the right are commonly cited as being consistent with gestational physiology.^19^ The upper reference intervals given here of 17 and 25 mm in the left and right kidneys respectively, are higher than the values of 12 and 16 mm derived from pregnant cohorts using ultrasound.^3^ Although comparison between published data is difficult due to variance in imaging technique, patient position, methods of assessment, and definitions of dilatation, this study suggests that ultrasound reference intervals should not be used for the interpretation of renal pelvis diameter in MRI.

The mean kidney volumes reported here are within the reported normal reference intervals for non-pregnant women on MRI and are lower than those derived from second-trimester ultrasound measurements.^10^ The finding of no difference in renal volume between the left and right kidney in pregnancy^10^ and an increase in kidney volume with advancing gestation are consistent with previous ultrasound studies in pregnancy,^2,17^ although the increase in kidney volume reported here is multidimensional, occurring without substantial increase in interpolar length. The increased accuracy and repeatability of estimations of kidney volume by MRI compared to ultrasound support the gestational morphology reported in this study.^7^

The primary weakness of this study is that the maternal renal MRI images used were captured incidentally rather than using dedicated renal MRI sequences. Imaging planes and acquisition were therefore not standardised for the kidney and the present findings must be interpreted in this context. A further weakness is that as the MRI examinations in this study were performed primarily in the second trimester, meaning the results may not be generalisable to other trimesters of pregnancy.

In this study, renal tract dilatation was reported as an incidental finding in approximately one in five healthy pregnant women undergoing fetal imaging. Following axial reformatting, 8% of right kidneys and 45% of left kidneys had dilatation of the renal pelvis greater than the commonly used upper reference limits of 20 and 8 mm for the right and left kidneys, respectively. The data presented here suggest that reference intervals for renal pelvis diameter in pregnancy should be reconsidered. Renal morphology data require confirmation in prospective studies using dedicated renal MRI image acquisition across gestation allowing the establishment of MRI-specific gestational reference intervals. The utility of kidney length and volume as markers of kidney function reserve, and the potential to utilise corticomedullary differentiation in the assessment of intrinsic renal disease in pregnancy, including the renal endotheliosis of pre-eclampsia, warrant further investigation.

## Figures and Tables

**Table 1:** Demographic and pregnancy outcome data.

Age (median [IQR])	34 [31–36]
Ethnicity	
White European	27 (64)
Mixed	2 (4.8)
Unknown^a^	13 (31)
Height, m (median [IQR])	1.65 [1.63–1.72]
Body mass index, kg/m^2^ (median [IQR])	23.1 [21.3–25.2]
Co-exiting conditions	
None	29 (69)
Asthma	3(7.1)
Hypermobility	2 (4.8)
Hypothyroidism	1 (2.4)
Polycystic ovarian syndrome	1 (2.4)
Rheumatoid arthritis	1 (2.4)
Sarcoidosis	1 (2.4)
Thrombocythaemia	1 (2.4)
Uterine fibroid	2 (4.8)
Unknown^a^	3 (7.1)
Obstetric complications	
Gestational diabetes	2 (4.8)
Hyperemesis gravidarum	1 (2.4)
Placenta praevia	1 (2.4)
Unknown^b^	5 (11.9)
Gestational age at delivery, weeks (median [IQR])	39.9 [39.1–40.7]
Birthweight, grams (median [IQR])	3,480 [3,250-3,700]
Birth outcome	
Livebirth	37 (88.1)
Unknown^a^	5 (11.9)

Results are presented as number (percentage) of the cohort unless specified. IQR, interquartile range.

a Antenatal care/delivery in a different hospital or data not specified.

b No clinical suspicion of renal involvement.

**Table 2:** Morphological measurements of left and right kidneys in pregnancy on magnetic resonance imaging and concordance (pooled data from left and right kidneys) between observer measurements.

Measurement	Left *n*	Left kidney (95% reference interval/limit)	Right *n*	Right kidney (95% reference interval/limit)	Difference between left and right kidneys [95% CI]	Concordance between observers [95% CI]	Mean difference in measurement between observers
Maximum interpolar length (cm)	42	10.9 (8.4-13.5)	42	10.4 (8.3-12.5)	þ0.5 [0.1 to 1.1]	0.98 [0.96 to 0.98]	0.10
Maximum renal pelvis dilatation (mm)	38	8.5 (16.7)	37	11.9 (25.1)	-3.6 [-6.1 to -0.9]	0.99 [0.99 to 1.00]	0.00
Kidney volume (cm^3^)	42	162.1 (83.9-236.9)	42	163.7 (94.2-234.1)	-1.6 [-17.0 to 13.9]	0.95 [0.93 to 0.97]	1.91
Corticomedullary differentiation ratio	42	0.84 0.77-0.91	34	0.84 (0.70-0.96)	0.0 [-0.02 to 0.02]	0.39 [0.20 to 0.55]	0.02

**Table 3:** Univariable linear regression of association between maternal characteristics and gestational age with maximum interpolar length and kidney volume.

Variable	*n*	Maximum interpolar length (cm)		Kidney volume (cm^3^)	
		Regression coefficient (95% confidence interval)	*p*-Value	Regression coefficient (95% confidence interval)	*p*-Value
Maternal age (years)	84	0.00 (-0.06 to 0.06)	0.952	0.25 (-1.67 to 2.17)	0.797
Maternal height (cm)	70	0.05 (0.01-0.09)	0.012	2.29 (1.13 to 3.44)	<0.001
Maternal BMI (kg/m^2^)	70	0.00 (-0.06 to 0.07)	0.965	0.17 (-1.91 to 2.25)	0.871
Gestational age at scan (days)	84	0.00 (-0.01 to 0.02)	0.531	0.45 (0.01 to 0.89)	0.043

The coefficient is a measure of the difference that can be attributed to that variable.
